# Off‐label drug use in hospitalized children: a prospective observational study at Gondar University Referral Hospital, Northwestern Ethiopia

**DOI:** 10.1002/prp2.304

**Published:** 2017-03-17

**Authors:** Yonas G. Tefera, Begashaw M. Gebresillassie, Abebe B. Mekuria, Tamrat B. Abebe, Daniel A. Erku, Nurahmed Seid, Habiba B. Beshir

**Affiliations:** ^1^ Department of Clinical PharmacySchool of PharmacyUniversity of GondarGondarAmharaEthiopia; ^2^ Department of PharmacologySchool of PharmacyUniversity of GondarGondarAmharaEthiopia; ^3^ Department of Pharmaceutical ChemistrySchool of Pharmacyuniversity of GondarGondarAmharaEthiopia; ^4^ Department of PharmaceuticsSchool of PharmacyUniversity of GondarGondarAmharaEthiopia

**Keywords:** Children, Ethiopia, Gondar, off‐label, pediatric

## Abstract

Most of the medications which are currently used for the treatment of childhood diseases are either not licensed or being prescribed outside the terms of the product license (off‐label prescribing). This study aimed at determining the extent of unlicensed and off‐label drug uses and associated factors in children hospitalized in Gondar University Referral Hospital, Northwest Ethiopia**.** An institution‐based prospective cross‐sectional study was employed from April 15 to July 15, 2016. A total of 243 pediatric patients admitted to Gondar university referral hospital were included in the study using simple random sampling method. Data were collected using structured questionnaire, and the data collected were entered and analyzed using Statistical Packages for Social Sciences (SPSS) version 20. From the total of 800 drugs prescribed, 607 (75.8%) were off‐label. Off‐label medicine use was frequently observed in antimicrobials (60.6%) followed by central nervous system drugs (14.3%). The extent off‐label prescribing was highest in age group of 6–13 years (30%). Inappropriate dosing and frequency (42.3%) were the most common reason for off‐label medicine use. Having other variables controlled, age group and undergoing surgical procedure remained to be significant predictors of off‐label prescribing in the multivariate regression analysis. Implementing evidence‐based approach in prescribing by generating more quality literatures on the safety profile and effectiveness of off‐label would improve the injudicious use of drugs in pediatric population.

**Abbreviation**
BNF: British national formulary
CAP: community‐acquired pneumonia
OR: odds ratio
PILs: Patient information leaflets

## Introduction

Realizing ideal drug therapy in pediatric population is a global concern for clinicians and regulatory agencies largely owing to the scarcity and low quality of evidence in safety and efficacy in pediatric population (Dunne 2007).

Use of medicines outside the specifications described in the license in terms of formulation, indications, contraindications constitutes off‐label and off‐licensed use. ‘Off‐label use’ is the use of a drug which has a marketing authorization, but is used for a condition, at a dose, via a route or for an age that is not listed in its product characteristics (Maria 2014). The off‐label and unlicensed use of drugs to treat children is widespread and occurs in medical and surgical wards, as well as in critically ill children (Sean et al. 1998). Medicines of major clinical importance including essential medicines are not tested and officially approved for use in this age group. Off‐label prescribing of drugs in pediatric population predisposes children to medication errors as doses must be calculated on an individual patient basis, often in the absence of appropriate dosing information from the pharmaceutical manufacturer. This is supported by different studies which reported that medication errors are three times more common in pediatric than adult patients (Sharif 2015; Hildtraud 2013).

The magnitude of off‐label prescribing is accounted to be between 15% and 60% in infants and 90% in neonates (Conroy et al. 2000; Cuzzolin et al. 2006; Jain et al. 2008; Bavdekar et al. 2009). The average number of drugs prescribed per child was 1.1 in Sweden 2007 (The National Board of Health and Welfare Health care registries 2008), and varied in other countries from 0.8 to 3.2 drugs per child per year. (Sanz et al. 1989; Clavenna and Bonati 2009). The most commonly prescribed off‐label drugs in children are antibiotics for systemic use, followed by drugs for the respiratory system and analgesics (Sanz et al. 1989; Sturkenboom 2008; Clavenna and Bonati 2009).

There are a bulk of published literatures around the globe regarding the off‐label and unlicensed drug use in pediatrics population (Sharif 2015; Sanz et al. 1989; Sean et al. 1998; Conroy et al. 2000; Cuzzolin et al. 2006; Jain et al. 2008; The National Board of Health and Welfare Health care registries 2008; Bavdekar et al. 2009; Clavenna and Bonati 2009; Hildtraud 2013). However, in Ethiopia, there is paucity of data on the nature and magnitude of off‐label and unlicensed drug use in children. The aim of present study was to determine the extent of off label drug use in children in hospitalized at Gondar University Referral Hospital, Northwest Ethiopia.

## Subjects and Methods

### Study design and setting

Institutional‐based prospective observational study was conducted on off‐label drug use in pediatric patients at Gondar University Referral Hospital (GURH) from April 15 to July 15, 2016. GURH is one of the oldest teaching hospitals in the country, located 738 km northwest of Addis Ababa, which is a capital city of Ethiopia. It has more than 400 beds and provides its services in various departments including pediatrics, surgery, gynecology, psychiatry, dermatology, dentistry, ophthalmology, pharmacy (outpatient, inpatient, antiretroviral, and emergency), medical laboratory and others. The pediatric ward has more than 100 beds and 25 pediatricians. All the pediatric patients aged between 0 and 18 years, receiving at least one medication and admitted in pediatric ward of GURH from April 15 to July 15, 2016 were included in the study.

### Data collection and management

The data collection was carried out by three of the researchers, through discussing each of the medicines with attending pediatrician and clinical pharmacist. Demographics, clinical characteristics, and medication usage were obtained from the medical records using predefined data abstraction format and through face to face interview. Off‐label use of drugs by pediatric patients were identified using British national formulary (BNF), Patient information leaflets (PILs) and the standard treatment guidelines of Ethiopia.

### Statistical analysis

The final data collection tool was ensured for completeness, and responses were entered into and analyzed by the Statistical Package for the Social Sciences (SPSS) software version 21.0 for Windows. Frequencies and percentages were used to express different sociodemographic characteristics and drug‐related variables. Univariate and multivariate logistic regression analysis were utilized to come up with factors associated (predictors) with off‐label prescribing. Associations with significance level of <0.20 (*P* < 0.20) in the univariate analysis were included in the multivariate logistic regression analysis. The results were adjusted for patients' demographic and clinical characteristics. Odds ratio (OR) with 95% confidence interval (95% CI) were also computed along with corresponding *P*‐value (*P* < 0.05).

### Ethical considerations

This study was approved by the ethical committee of University of Gondar and the Clinical Directorate of Gondar University Referral Hospital. Informed consent from the patients was also obtained before conducting this study. Participants' information obtained was kept confidential.

## Results

### Demographic and clinical characteristics

Out of the total 252 cases admitted in pediatric general ward of GURH over 3‐month period, 243 were included in the study, and 9 patients were excluded due to incompleteness making the response rate 96.4%. More than half (57.2%) of participants were males with a mean (SD) age of 4.54 ± 4.32 years. The most common age group was 0–1 years representing 28.8% of total sample size. The mean number of prescriptions per child was 4 with the standard deviation (SD) of 3 and 57.6% of patients pay full cost of their prescriptions (Table 1).

**Table 1:** Distribution of respondents by sociodemographic characteristics, GURH, 2016

Variable	Frequency (%)
Age	
0–28 days	32 (13.2)
1 month–2 year	59 (24.3)
2–6 years	70 (28.8)
6–13 years	64 (26.3)
13–18 years	18 (7.4)
Sex	
Female	104 (42.8)
Male	139 (57.2)
Area of Residence	
Urban	122 (50.2)
Rural	121 (49.8)
Unit of admission	
General	168 (69.1)
Critical	33 (13.6)
Neonatology	25 (10.3)
Surgery	17 (7.0)
Length of stay, in days	
0–7	184 (75.7)
7–14	48 (19.75)
>14	11 (4.52)
Surgery	
Yes	30 (12.3)
No	213 (87.7)
Number of prescription per patient	
1–3	129 (53.09)
4–6	78 (32.10)
>7	36 (14.8)

The majority of patients suffered from community acquired pneumonia 49 (12%), Severe acute malnutrition 44 (10.7%), and skin and soft tissue infections 43 (10.5%). Majority of patients 184 (75.7%) stay in hospital for 0–7 days (Table 2).

**Table 2:** Most common diagnosis in pediatrics ward of GURH, 2016

Diagnosis	Number (%)
Community acquired pneumonia	49 (12)
Severe acute malnutrition	44 (10.7)
Skin and soft tissue infections	43 (10.5)
Sepsis	36 (8.8)
Pyogenic meningitis	26 (6.3)
Heart failure	25 (6.1)
Surgical and intraabdominal infections	23 (5.6)
Seizure	19 (4.6)
Acute gastroenteritis	17 (4.2)
Cancer and neutropenic fever	14 (3.4)
Others	114 (27.8)
Total	410 (100)

### Nature of off‐label prescribing

**Figure 1:**
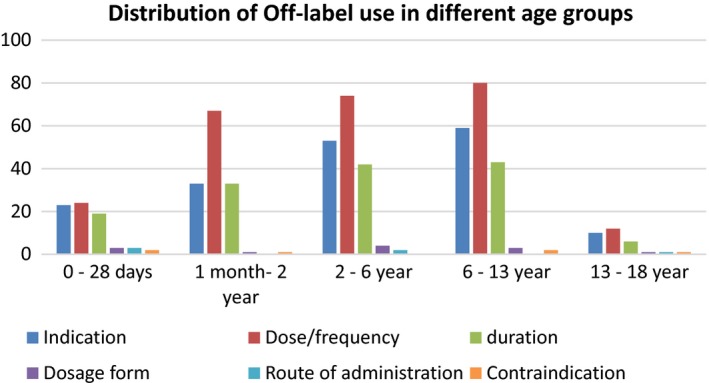
Drug use by in‐label and off‐label medicine use in pediatrics ward of GURH, 2016.

Of the 243 patients included in the study, About 190 (78.1%) children were prescribed at least one off‐label medicine. Patients received on average ± SD of 3.26 ± 2.41 off‐label medicines ranging from 1 to 7. A total of 800 medications were prescribed during hospitalization with an average of 3.3 prescriptions per patient. Among these, 607 (75.8%) medicine uses were classified as off‐label when its usage was validated with PIL and STG, for six different off‐label categories. The most common cause of off‐label prescribing (43%) was due to higher dose/frequency followed by indication (30.1%) and duration (23.5%). (Fig. 1).

### Off‐label prescribing in different Age groups

Off‐label medicines prescribed were distributed in different age groups as shown in Figure 1. The extent off‐label prescribing was highest in age group of more than 6–13 years, followed by age group of 2–6 years. Off‐label use in dose and indication was high among the patients in age of 6–13 years.

### Off‐label drug use by pharmacologic classes

Overall, off‐label medicine use is highest (60.6%) for products belonging to the general antimicrobials, followed by drug classes acting on central nervous system (14.3%) and cardiovascular system (8.6%). In contrast, ophthalmologic and blood‐forming agents were the least group of drugs (0.3%) among the off‐label prescriptions. Off‐label dosage form and off‐label route of administration is higher in the class of drugs acting on central nervous system. In all other classes off‐label dosing is dominating (Table 3)**.**

**Table 3:** Predictors of Off‐label drug use in pediatrics ward of GURH, 2016

Variables	Off‐label use (*n* = 243)		COR (95% CI)	AOR (95% CI)	*P‐* value
	Yes (%)	No (%)			
Age					0.022^a^
0–28 days	24 (12.6)	219 (87.4)	1.50 (0.42–5.31)	1.85 (0.26–13.37)	
1 month–2 year	46 (14.2)	197 (75.8)	1.77 (0.56–5.63)	2.23 (0.64–7.77)	
2–6 years	51 (10.8)	192 (79.2)	1.34 (0.44–4.08)	1.60 (0.49–5.23)	
6–13 years	57 (13.5)	186 (76.5)	4.07 (1.16–14.29)	4.703 (1.24–17.76)	
13–18 years	12 (4.9)	231 (95.1)	1	1	
Sex					0.507
Female	79 (32.5)	164 (67.5)	1	1	
Male	111 (45.7)	132 (54.3)	1.25 (0.68–2.31)	1.25 (0.64–2.43)	
Area of Residence					0.119
Rural	99 (40.8)	144 (59.2)	1	1	
Urban	91 (37.5)	152 (62.5)	0.65 (0.35–1.21)	0.57 (0.28–1.16)	
Unit of admission					0.467
General	126 (51.9)	117 (48.1)	0.95 (0.35–2.53)	0.80 (0.12–5.48)	
Critical	30 (12.6)	213 (87.6)	3.16 (0.70–14.16)	2.35 (0.24–23.45)	
Neonatology	15 (6.2)	228 (93.8)	2.37 (0.42–13.46)	0.82 (0.06–12.00)	
Surgery	19 (7.8)	224 (92.2)	1	1	
Length of stay, in days					0.359
0–7	139 (57.2)	104 (42.8)	0.31 (0.04–2.48)	0.36 (0.40–3.21)	
7–14	41 (16.9)	202 (83.1)	0.59 (0.06–5.32)	0.61 (0.06‐6.26)	
>14	10 (4.1)	233 (95.9)	1	1	
Surgery					
Yes	28 (11.5)	215 (88.5)	4.41 (1.01–19.14)	4.27 (0.86–21.25)	0.002^a^
No	162 (66.7)	81 (33.3)	1	1	

a *P* < 0.05.

**Figure 2:**
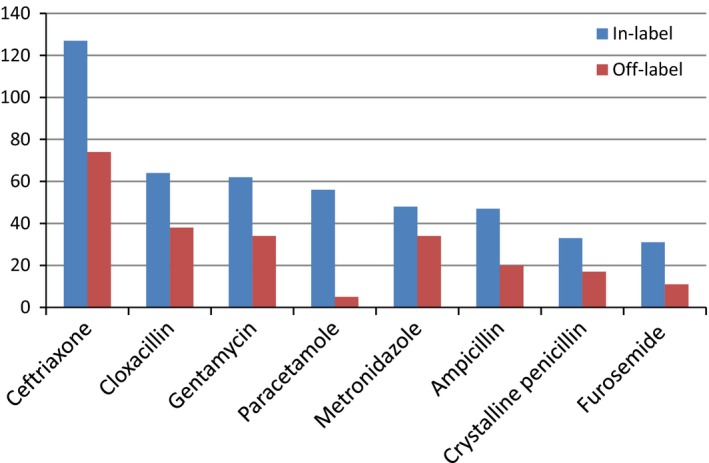
Comparison of in‐label and off‐label use of some of commonly prescribed drugs in pediatrics ward of GURH, 2016.

The most commonly off‐ label prescribed drug was ceftriaxone 74 (20.7%), followed by cloxacillin 38 (10.6%), gentamycin 34 (9.52), and paracetamol 5 (1.4%) (Fig. 2).

### Predictors of off‐label drug use

Having other variables controlled, age group and undergoing surgical procedure were remained to be significant in the multivariate regression analysis. Accordingly, patients in the age of 6–13 years (Adjusted odds ratio (AOR) = 4.703, CI = 1.24–17.76) were more likely to receive off‐label medicines than any other age group. Similarly, patients undergoing surgical procedure (AOR = 4.273, CI = 0.86–21.25) received substantially high amount of off‐label medicines.

## Discussion

Unlike the developed world, the importance of drug safety and efficacy in pediatric population has been gaining less attention in the developing countries like Ethiopia despite the widespread use off label and unlicensed drugs. To the best of our knowledge, this is the first study done in Ethiopia regarding the off‐label use of drugs in pediatric population.

According to the finding of our study, the overall prevalence of off‐label medicine use is 75.8%, which is similar with the study reported in Finland (76%) and United States of America with a percentage of 78.7% (Sean et al. 1998; Turner et al. 1998). However, this was much higher compared with study conducted in Germany (30%)(Hildtraud 2013) and the Netherlands (44%). (Mukattash et al. 2012). The rate of off‐label drug use is higher among males than females and the highest rate was observed in age group of 6–13 years, which is comparable with the population‐based report from Germany (Hildtraud 2013). This might have resulted due of lack of pediatric labeling, which the most common cited reasons for off‐label drug use in nearly all of the studies (Kimland 2010). Some of the prescriber physicians treat adolescents as adults, which is not fully justified (Schirm 2003).

The high rate of off‐label use could also be due to lack of harmonization between pediatric documentation in the existing literature evidence and the authorized drug label, which affects the physicians' prescribing practice. There is a need for expert groups devoted to pediatric drug treatment within the organization of the Drug and Therapeutic Committees, which could continuously process new literature data and convey relevant information to prescribing physicians. Only such focused and coordinated actions would make sure that children's right to safe, cost‐effective, and quality medicines would be realized.

The therapeutic drug categories that are most commonly used off‐label in our hospital were general antimicrobials and drugs acting on central nervous system. This finding was in agreement with study conducted in Sweden. However, in the majority of studies, the highest proportion of off‐label drug use in children was topically administered drugs such as dermatological and eye drops (Kimland and Odlind 2011). Several studies had shown high rate of off‐label prescribing in respiratory (Jong et al. 2004), antibiotics (Ekins‐Daukes et al. 2003), analgesics (Conroy and Peden 2001), and antiepileptics (Novak et al. 2005). Ceftriaxone, cloxacillin, and gentamyicin were the top three medications which are frequently used in off‐label manner. This finding was different compared to the report from Sweden, which reported morphine, paracetamol and salbutamol as the most common off‐label medications used. The variation could be explained by the fact that the most prevalent diseases, prescribing trend and availability of medications varies across different countries.

The most frequent category of off‐label use in our study was inappropriate dosing and/or frequency (42.3%). Similar findings were reported from the survey in pediatric ward of European countries, Germany, Brazil and Scotland (Conroy et al. 2000; Daukes et al. 2004; Porta et al. 2010; Hildtraud 2013). Off‐label medicine use, such as under‐ and/or overdosing, could bear the risk of potential health hazards. Inappropriate dosing is of particular concern for antibiotic use with respect to the development of resistances (McDonnell 2008; Dryden et al. 2011). If medication is underdosed, there may be no therapeutic benefit but carries a risk for ADRs. Among all pediatric patients surveyed, those receiving care for the most frequent diagnosis, which is community acquired pneumonia (CAP), are the leading recipients of off‐label medicines.

This finding was different from study conducted in Brazil, which reported that the leading recipients of off‐label medicines were those receiving oncologic care. In our study, age group of 6–13 years and undergoing surgical procedure were independent determining factors for the probability of off‐label medicine use. This finding was in agreement with reports from USA and Germany (Shah 2007; Hildtraud 2013).

World Health Organization (WHO) adopted “Better Medicines for Children” to improve medicine safety in the pediatrics and highlighted its concern on off‐label medicines (Hoppu and Anabwani 2012). The strict drug approval procedure is the way to ensure quality data on quality, safety, and efficacy for different pediatrics medications. Even though there are continuous policy amendments in this area, the problem of quality evidence in pediatric clinical trials is universal (Selvarajan et al. 2013). The pharmaceutical companies should also have appropriate pediatric labeling.

The study has some limitations that should be taken into account while interpreting the results. Because the study is conducted in only one referral hospital, the results found regarding Off‐label use may not be representative of patients outside Gondar region. A larger scale and multicentered study that includes more diverse patients is needed to provide more accurate findings.

## Conclusion

The finding of our study revealed that magnitude of off‐label prescribing in pediatric inpatients is considerably high. Dosing and/frequency discrepancy were identified as main contributor to off‐label prescribing, which predisposes children to the occurrence of side effects without a therapeutic effect. Particularly for antibiotics, the development of resistances is fostered when low doses/subtherapeutic doses are given. Age group and undergoing surgical procedure were identified as strong predictors of off‐label use. Implementing evidence‐based approach in prescribing drugs by generating more quality literatures on the safety profile and effectiveness of off‐label would improve the injudicious use of drugs in pediatric population. Further studies are needed to examine the situation in a national level.

## Disclosure

All authors confirmed no conflict of interest in this paper.
